# Substrate-Driven Convergence of the Microbial Community in Lignocellulose-Amended Enrichments of Gut Microflora from the Canadian Beaver (*Castor canadensis*) and North American Moose (*Alces americanus*)

**DOI:** 10.3389/fmicb.2016.00961

**Published:** 2016-06-21

**Authors:** Mabel T. Wong, Weijun Wang, Michael Lacourt, Marie Couturier, Elizabeth A. Edwards, Emma R. Master

**Affiliations:** Department of Chemical Engineering and Applied Chemistry, University of Toronto, TorontoON, Canada

**Keywords:** 16S rRNA pyrosequencing, lignocellulose degradation, microbial community composition, microbial enrichment, microbial convergence, digestive microflora, beaver dropping, moose rumen

## Abstract

Strategic enrichment of microcosms derived from wood foragers can facilitate the discovery of key microbes that produce enzymes for the bioconversion of plant fiber (i.e., lignocellulose) into valuable chemicals and energy. In this study, lignocellulose-degrading microorganisms from the digestive systems of Canadian beaver (*Castor canadensis*) and North American moose (*Alces americanus*) were enriched under methanogenic conditions for over 3 years using various wood-derived substrates, including (i) cellulose (C), (ii) cellulose + lignosulphonate (CL), (iii) cellulose + tannic acid (CT), and (iv) poplar hydrolysate (PH). Substantial improvement in the conversion of amended organic substrates into biogas was observed in both beaver dropping and moose rumen enrichment cultures over the enrichment phases (up to 0.36–0.68 ml biogas/mg COD added), except for enrichments amended with tannic acid where conversion was approximately 0.15 ml biogas/mg COD added. Multiplex-pyrosequencing of 16S rRNA genes revealed systematic shifts in the population of *Firmicutes*, *Bacteroidetes*, *Chlorobi*, *Spirochaetes*, *Chloroflexi*, and *Elusimicrobia* in response to the enrichment. These shifts were predominantly substrate driven, not inoculum driven, as revealed by both UPGMA clustering pattern and OTU distribution. Additionally, the relative abundance of multiple OTUs from poorly defined taxonomic lineages increased from less than 1% to 25–50% in microcosms amended with lignocellulosic substrates, including OTUs from classes *SJA-28*, *Endomicrobia*, orders *Bacteroidales*, *OPB54*, and family *Lachnospiraceae*. This study provides the first direct comparison of shifts in microbial communities that occurred in different environmental samples in response to multiple relevant lignocellulosic carbon sources, and demonstrates the potential of enrichment to increase the abundance of key lignocellulolytic microorganisms and encoded activities.

## Introduction

Lignocellulose in agricultural and forest residues, as well as energy crops, is considered as an important renewable resource for the production of bioenergy, liquid biofuels, and specialty chemicals. As the main component of plant cell walls, lignocellulose is largely composed of polysaccharides (cellulose, hemicellulose, and pectin) and lignin, with varying chemical compositions and structures depending on plant species, tissue, and cell type (Harris and Stone, 2009). Wood fiber typically contains a higher lignin content and hemicelluloses with chemical structures distinct from those found in grasses. Fungi and bacteria are the dominant organisms responsible for lignocellulose biodegradation and encoded enzymes offer advantages in lignocellulose processing particularly when (i) converting lignocellulose into fermentable intermediates, (ii) synthesizing high-value chemicals from specific lignocellulose components, and (iii) handling residual biomass with high water content, which are less amendable to processing through thermo-chemical options.

Metagenomic analysis of microbial communities that degrade lignocellulose has been motivated by decreasing DNA sequencing costs, along with the rich repertoire of CAZymes encoded by gut microflora. Such efforts have included the analysis of metagenomes obtained from foregut of Tammar wallaby (Pope et al., 2010), mid-gut of wood-feeding Asian longhorn beetles (Scully et al., 2013), hindgut of termite (Warnecke et al., 2007), as well as the rumen of ox (Brulc et al., 2009), cow (Hess et al., 2011), yak (Dai et al., 2012), and reindeer (Pope et al., 2012). Corresponding analyses have identified thousands of new genes predicted to encode enzymes relevant to lignocellulose conversion. For instance, metagenomic analysis of the cow rumen alone led to over 27,000 new candidate CAZymes (Hess et al., 2011). In an effort to identify genes likely to encode enzymes optimized for transforming wood fiber, Scully et al. (2013) applied hierarchical cluster analysis of Pfam abundances to compare the gut metagenome of a wood-boring pest, *Anoplophora galbripennis*, to 19 herbivore-related metagenomes (Scully et al., 2013). Distinct clusters representing different herbivore biome-types were identified, including herbivore gut communities, fungal gallery communities, and communities associated with insects that feed on heartwood.

In contrast to grass-fed mammalian herbivores, North American moose (*Alces americanus*) and Canadian beavers (*Castor canadensis*) are iconic Canadian foragers of coniferous and deciduous trees in riparian zones of the boreal mixed-wood forests (Hood and Bayley, 2009). North American moose is the largest browsing ruminant of the deer family *Cervidae* (Ishaq and Wright, 2012), while Canadian beavers represent one of the largest and ecologically most distinct rodent species with a monogastric digestive system, whose dietary subscription shifted from omnivory to obligate herbivory (Horn et al., 2011). With wood biomass being a significant part of the diet, the microbial communities within the digestive system of these Canadian mammals are likely to include lignocellulose-degrading bacteria. Recent studies report lignocellulose-degrading bacterial lineages among the gut microbes from moose (Ishaq and Wright, 2014), which resemble those residing in the termite hindgut (Ishaq and Wright, 2012). Preliminary data also suggest cellulolytic/xylanolytic activities in the lower gut of beavers (Gogola et al., 2011).

Enrichment of microbial communities on selected lignocellulose substrates could augment the fraction of most pertinent lignocellulose degraders and encoded activities. For example, feeding termites with grasses enriched *Clostridiales incertae sedis* and *Spirochaetaceae* lineages of *Firmicutes* in their hindgut populations, whereas feeding with wood fiber proliferated members across several phyla, including *Bacteroidetes*, *Elusimicrobia*, *Firmicutes*, *Plantomycetes*, *Proteobacteria*, *Spirochaetes*, and *Verrumicrobia* (Huang et al., 2013). Similarly, fecal microbiomes obtained from cattle fed with unprocessed grain were enriched with bacteria belonging to the *Ruminococcaceae* order, whereas those obtained from cattle fed with forage or processed grain were enriched in bacteria belonging to the *Prevotella* genus (Order: Bacteroidales; Shanks et al., 2011). Notably, specific phyla were enriched in nearly all lignocellulose-degrading gut microflora analyzed to date, including *Firmicutes* (*Bacilli*, *Clostridia*), *Proteobacteria*, *Bacteroidetes*, *Chloroflexi*, and *Actinobacteria.* However, most enrichment studies have been performed *in situ*, and so are confounded by the presence of additional glycan sources, including mucin glycans produced by the host (Koropatkin et al., 2012; Tailford et al., 2015). Alternatively, *ex situ* enrichment of microbial communities on lignocellulosic carbon sources could uncover microbial lineages that are quintessential to degrading specific biomass components.

Here, we directly compared shifts in microbial profiles that result from long-term enrichment (>3 years) of digestive microflora from the Canadian beaver and North American moose, on four lignocellulosic carbon sources: C, CT, CL, and PH. These amendments represented increasingly complex and potentially inhibitory carbon sources. For example, inhibition of methanogenic activity by tannic acids is well known (Bhatta et al., 2009), while PH typically contains mixed-wood extractives, organic acids, furan derivatives, and lignin that can inhibit microbial activity, including methanogenesis (den Camp et al., 1988; Sierra-Alvarez and Lettinga, 1991; Mills et al., 2009). Aside from monitoring metabolic activities through biogas yield from each enrichment, pyrotag sequencing was performed to characterize shifts in microbial communities that would suggest specialization and expression of distinct lignocellulolytic activities.

## Materials and Methods

### Ethics Statement

A hunting license was authorized by the Ministry of Natural Resources and Forestry under Government of Ontario, Canada, for the collection of moose rumen sample.

### Collection of Beaver Droppings and Moose Rumen Samples

Beaver droppings were collected in November 2009 with assistance from the Ontario Ministry of Natural Resources. Collection sites included mixed boreal forests near Parry Sound, Mount Albert, and Mississauga. MR fluid samples were collected by hunters on October 10, 2009 at a location just west of Nabakwasi Lake (GPS: 47.5612 N, 81.4504 W), about 100 km north of Sudbury, Ontario. All samples were stored on ice during transport, and then transferred to –20°C upon arrival at the University of Toronto.

### Carbon Sources and their Chemical Oxygen Demand

Amended carbon sources included (i) microcrystalline cellulose (C; (Avicel PH101, Sigma–Aldrich, MO, USA), (ii) cellulose (Avicel) + lignosulphonate (CL; Tembec Industries Inc., QC, Canada), (iii) cellulose (Avicel) + tannic acid (CT, Sigma–Aldrich, MO, USA), and (iv) steam exploded PH (SunOpta Inc., Canada; October, 2009). To determine the stoichiometric maximal biogas yield (Symons and Buswell, 1933) of these lignocellulose substrates (in ml biogas/mg COD), the chemical oxygen demand (COD) was determined as described by the APHA standard method 5220D using potassium dichromate as an oxidizing agent (Clesceri, 1998) (**Supplementary Table S1**).

### Setup and Maintenance of Anaerobic Enrichments

Based on the protocol established previously by Grbić-Galić and Vogel (1987) and Edwards and Grbić-Galić (1994), sulphide-reduced mineral medium (pH 7.0) was prepared, autoclaved at 121°C for 20 min and purged with 80% N_2_, 20% CO_2_ gas mixture to maintain methanogenic consortia. The collected BD and MR were thawed and mixed separately to ensure sample homogeneity. To begin each anaerobic cultivation, the prepared mineral medium, environmental inoculum, and autoclaved lignocellulosic carbon sources were introduced into an anaerobic glove-bag (medium tape seal AtmosBag, Aldrich; Edwards and Grbić-Galić, 1994). Approximately 15 ml of BD or MR inoculum was then transferred to separate 160 ml Wheaton glass serum bottles, which were subsequently amended with selected lignocellulosic substrates (average 36.1 mg COD equivalent) and 45 ml of mineral medium (**Supplementary Table S2**). Corresponding cultures were prepared in triplicate and sealed with butyl rubber stoppers before being incubated anaerobically at 36°C; biogas production was then monitored at regular intervals using a pressure transducer (Omega PX725 Industrial Pressure Transmitter, Omega DP24-E Process Meter).

When biogas production ceased, each microbial community was transferred to a new bottle and amended with fresh anaerobic medium and lignocellulose carbon source (approximately 170 mg COD; **Supplementary Table S2**). Transfer by dilution was performed in the first four phases, wherein approximately 1/3 of the entire enrichment was transferred to a new bottle and topped up with fresh carbon source and medium to the original volume. To subsequently increase the density of lignocellulose-active communities and remove accumulated metabolic wastes, subsequent enrichments were centrifuged at 6000 rpm for 15 min at 24°C, and then the spent cultivation medium was replaced with fresh mineral medium and carbon source. The amounts of lignocellulose substrates added to the microcosm bottles and duration across the enrichment phases are summarized in **Supplementary Tables S2** and **S3**.

### DNA Extraction from Inocula and Anaerobic Enrichments

Following 3 years of cultivation, comprising over 10 enrichment phases (**Supplementary Table S2**), 10 ml of each culture derived from MR and BD were harvested at early stationary phase of biogas production by centrifugation at 10,000 rpm for 15 min at 4°C. Total community DNA was extracted from corresponding cell pellets using the QIAamp DNA Stool Mini Kit (Qiagen, Hilden, Germany). DNA was also extracted from ∼1 g (wet weight) of original BD and MR inocula that had been stored at –80°C. The manufacturer’s instructions were followed, including 5 min incubation at 95°C to improve DNA recovery. The extracted metagenomic DNA was then quantified using a Nanodrop 2000 spectrophotometer (Thermo Scientific, MA, USA) and stored at –80°C.

### Multiplex-Pyrosequencing of 16S rDNA

Polymerase chain reaction (PCR) was used to amplify the V5-8 hypervariable region of 16S rRNA genes for multiplex-pyrosequencing. A set of 10-nt barcodes designed by Roche was incorporated to the 926 Forward (5′-AAACTYAAAKGAATTGACGG) and 1392 Reverse (5′-ACGGGCGGTGTGTRC) primers for multiplexing (**Supplementary Table S4**) (DeAngelis et al., 2012). PCR reactions were performed using *Taq* DNA polymerase in 2X PCR Master Mix (Thermo Scientific, MA, USA) with the following conditions: (i) initial denaturation at 95°C for 3 min and (ii) 35 cycles of 95°C for 30 s, annealing at 55°C for 45 s, and elongation at 72°C for 90 s, followed by (iii) a final extension at 72°C for 10 min. Amplicons were purified using the GeneJET PCR Purification Kit (Thermo Scientific, MA, USA) and quantified using agarose electrophoresis and a Nanodrop 2000 spectrophotometer (**Supplementary Table S4**), before being sent to the Génome Québec Innovation Centre for precise quantification using PicoGreen assay (Thermo Scientific, MA, USA) and sequencing using a 454 GS FLX platform (454 Life Sciences – a Roche Company, Branford, CT, USA).

### Data Analyses of Pyrosequences

Pyrosequencing flowgrams were converted into sequence reads and quality scores using Roche 454 Life Science propriety software^1^ and then analyzed by Quantitative Insights Into Microbial Ecology (QIIME) 1.8.0 (Caporaso et al., 2010). Sequences were quality checked by filtering those with (i) quality scores less than 25, (ii) lengths less than 200 bp, and (iii) longer than 1000 bp. Uclust then clustered high-quality 16S rRNA sequences into OTUs at the threshold of 97% sequence similarity (Edgar, 2010). Representative sequences from each OTU were aligned to the 16S rRNA sequences archived in the Greengenes Database for taxonomic inference (DeSantis et al., 2006). Based on the OTU tables, alpha diversity analysis was used to generate rarefaction plots in QIIME. Using the same program package, hierarchical clustering using UPGMA was also calculated to interpret the beta diversity distance matrix. Finally, double dendrogram and Venn diagrams were generated using R scripts^2^ to visualize the taxonomic affiliation and the distribution of core OTUs among the enrichments.

The raw sequence reads are submitted to National Center for Biotechnology Information (NCBI) under the BioprojectID PRJNA298982.

## Results and Discussion

### Establishment of Biogas-Producing Microbial Enrichments

**FIGURE 1:**
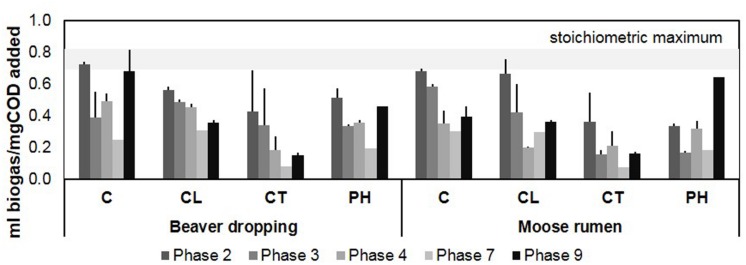
**Biogas production profile of microcosms fed with various lignocellulosic carbon sources for over 3 years.** The range of stoichiometric maximum biogas yield is shown in the gray band to provide a reference for the conversion extents of the fed substrates in the microcosms [see supplemental methods for the calculation based on Buswell’s formula (Symons and Buswell, 1933)]; error bars indicate standard deviation; *n* = 3. C, cellulose; CL, cellulose + lignosulphonate; CT, cellulose + tannic acid; PH, poplar hydrolysate.

Anaerobic enrichments were established and methanogenic activity was sustained over 10 feedings for 3 years on four different lignocellulosic substrate mixes (**Supplementary Table S2**). Over the enrichment phases, the average volumes of biogas produced per amount of COD added decreased initially and then increased by the ninth growth phase, suggesting acclimatization to each lignocellulosic carbon source by 154–171 weeks of enrichment (**Figure 1**, **Supplementary Table S3**). At the growth phase just prior to DNA extraction (i.e., phase 9), the extent of carbon source conversion for BD enrichments were: C (0.68 ml biogas/mg COD added), PH (0.46 ml biogas/mg COD added), CL (0.36 ml biogas/mg COD added), and CT (0.15 ml biogas/mg COD added). By comparison, the extent of carbon source conversion for MR enrichments was: PH (0.64 ml biogas/mg COD added), C (0.39 ml biogas/mg COD added), CL (0.36 ml biogas/mg COD added), and CT (0.16 ml biogas/mg COD added). Tannic acid consistently inhibited biogas production. The impact of tannic acid is consistent with previous studies, which report that the phenolic hydroxyl groups of tannic acid can complex with proteins, metal ions, amino acids, and polysaccharides (Makkar, 2003), thereby inhibiting enzyme action and uptake of essential carbon sources and metal ions (Asiegbu et al., 1995; Bhatta et al., 2009).

### Biodiversity Indices in Enrichment Cultures

Overall, 179,801 high-quality reads were retained for downstream analyses of community structure, richness, and diversity estimators (**Supplementary Table S5**). Despite extraction of high-quality DNA and successful PCR amplification, few reads (less than 15) were retrieved for BD cultivations enriched on CT (data not shown), and so this dataset was removed from downstream analyses. In total, recovered sequences were assigned to 5800 unique OTUs at 97% similarity threshold. In the MR enrichments, the decrease in Chao I (richness) and Shannon (diversity) indices compared to the original inoculum were consistent with enrichment of microorganisms best suited to transform amended carbon sources (**Supplementary Figure S1**). A similar trend was reported recently in soil microbiota following enrichment with wheat straw (Jimenez et al., 2014). In contrast, in the BD enrichments, the comparatively low Chao I index of the inoculum likely reflects the dominance of a soil species as described in the next section. Consistent with this interpretation of Chao I richness, the Simpson’s diversity index was highest for the BD and MR inoculum samples compared to corresponding enrichments. While the time span between defecation and sampling was unclear (as is the case for most fecal studies of wild animals), sample collection from beavers in the wilderness rather than captivity overcomes potential human interference to the gut microbiota. For example, loss in microbial diversity upon captivity has been reported in closely related woodrats (Kohl et al., 2014). With greater loss in microbial diversity in the diet specialist (e.g., Stephens’ woodrat which consumes a diet of 60–95% juniper) than the diet generalist (e.g., white-throated woodrat consumes actus, yucca, juniper, other shrubs and grasses), where the original microbiota could not be restored despite the provision of a natural diet.

**FIGURE 2:**
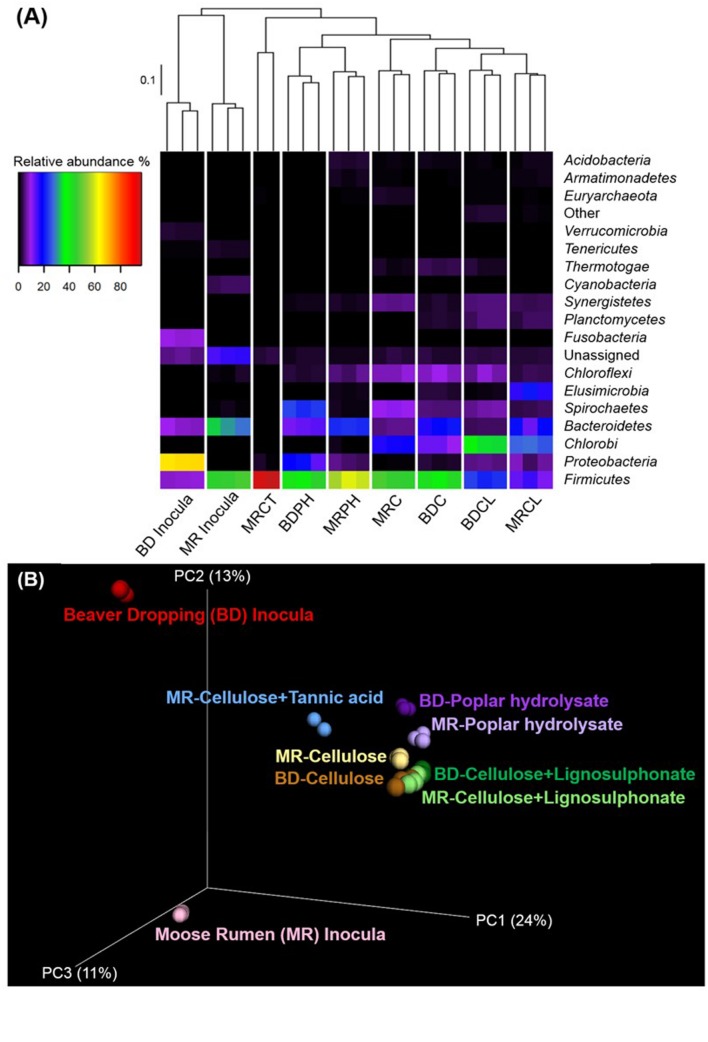
**Substrate-based clustering of lignocellulose-active microbial communities in beaver dropping and moose rumen, and their corresponding enrichment cultures.** **(A)** Heatmap with UPGMA clustering and relative abundances of microbial phyla (≥ 1.0% in at least one sample). **(B)** Three-dimensional Unifrac PCoA plot. BD, beaver dropping; C, cellulose; CL, cellulose + lignosulphonate; CT, cellulose + tannic acid; MR, moose rumen; PH, poplar hydrolysate.

A high level overview of the amplicon sequencing data revealed that samples from triplicate microcosms for a given enrichment condition clustered most closely, providing confidence in the reproducibility of the analysis (**Figure 2A**). Moreover, the inoculum samples were most divergent from subsequent enrichment cultures, clearly revealing substrate-driven convergence of microbial communities by both UPGMA and Unifrac clustering (**Figures 2A,B**). In the following sections, we first discuss community shifts in the BD samples, and then those in the MR samples, and finally a holistic view of the emergent OTUs that are shared in the enrichment microcosms.

### Impact of Lignocellulosic Substrates on Microbial Communities Originating from Beaver Droppings

**FIGURE 3:**
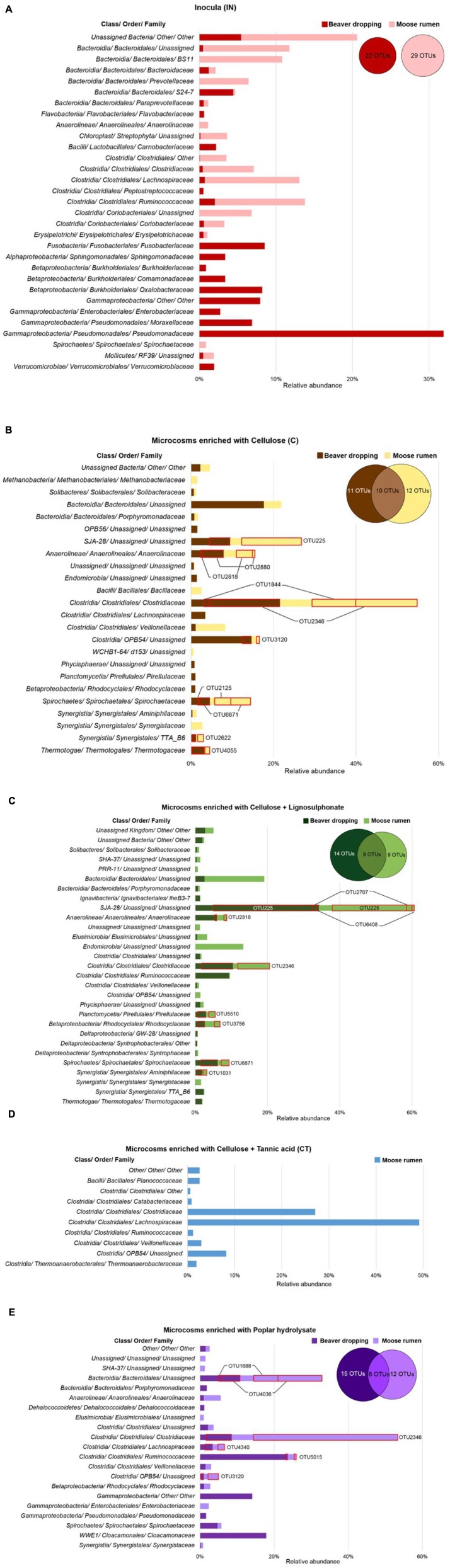
**Relative abundances of microbial families (≥ 1.0% in at least one sample) in beaver dropping and moose rumen, and their corresponding enrichment cultures.** **(A)** Inocula and microcosms fed with **(B)** cellulose, **(C)** cellulose + lignosulphonate, **(D)** cellulose + tannic acid, and **(E)** poplar hydrolysate. The distributions of OTUs (≥ 0.5% in at least one sample) are shown in the Venn diagram; shared OTUs are highlighted with red.

Upon enrichment on lignocellulosic substrates under strictly anaerobic methanogenic conditions, we observed a dramatic decrease in the microbes belonging to the *Proteobacteria* (**Figure 2A**). Specifically, the relative abundance of *Proteobacteria* diminished from approximately 62% in BD inoculum (sum of all *Proteobacteria* shown in **Figure 3A**, **Supplementary Data S1**) to between 1 and 17% in corresponding enrichments (**Figures 3B,C,E**), where highest numbers remained in cultures enriched on PH (**Figure 3E**). Most of the *Proteobacteria* present in the inoculum belonged to the genus *Pseudomonas* (approximately 30%; **Figure 3A**), which are ubiquitous soil facultative bacteria, and notably also comprise species with ability to detoxify lignocellulosic hydrolysates (Lopez et al., 2004). Although enrichment on PH retained a comparatively high fraction of *Proteobacteria*, the largest group in that enrichment was assigned to the genus *Gammaproteobacteria* (14%; **Figure 3E**). Notably, *Gammaproteobacteria* were previously identified in other biomass-degrading communities, including a cellulose-degrading marine biofilm and a wheat straw-degrading microbial consortia (Edwards et al., 2010; Jimenez et al., 2014). Similarly, the *Fusobacteriaceae* family represented 8.5% of the BD inoculum (**Figure 3A**), but were not detected in any of corresponding enrichments (**Figures 3B,C,E**). This family comprises microaerophilic to obligate anaerobes that can ferment carbohydrates and amino acids into various organic acids in anaerobic environments, including oral, gastrointestinal lining of mammals and anaerobic sediments (Olsen, 2014).

The impact of microbial enrichment was further illustrated by the detection of microbial phyla in enrichments that were not detected in the BD inoculum given their low abundance. For example, *Chlorobi* in the C and CT enrichments represented over 10 and 35% of corresponding communities (**Figures 3B,C**), even though this phylum was not detected in the original inoculum. An unassigned member of the uncultured class *SJA-28* constituted nearly 10% of C enrichments and 35% CL enrichments (**Figures 3B,C**), while comprising 83% and 95% of the *Chlorobi* phylum in these respective cultures. Enrichment on lignocellulosic substrates also led to the detection of *Spirochaetes* and *Chloroflexi* in enrichments originating from BD. Most notably among the *Spirochetaceae*, bacteria belonging to the genus *Treponema* contributed approximately 5% of all enrichments (**Figures 3B,C,E**), whereas the genus *W22* from the *Cloacamonaceae* family comprised over 17% of the microbial community enriched on PH (**Figure 3E**). *Treponema* acetogens were previously identified in the termite gut microbiome, and were predicted to encode glycoside hydrolases targeting cellulose and xylan (Warnecke et al., 2007).

Among the members of *Bacteroidetes*, family S24-7 made up 4% of the community in the BD inoculum (**Figure 3A**), while an unassigned lineage contributed up to 18% in enrichments established on cellulose (**Figure 3B**), and 11% of enrichments established on PH (**Figure 3E**). Notably, uncultured *Bacteroidetes* lineages dominate numerous lignocellulose-degrading communities. For example, recent metagenomic studies of microbiomes from human gut and reindeer rumen revealed high abundance of polysaccharide utilization loci-like systems originating from *Bacteroidetes* (Martens et al., 2009; Pope et al., 2012). These gene clusters encode CAZymes as well as transport proteins for glycan hydrolysis and uptake and represent a rich reservoir of new lignocellulolytic activities (Terrapon et al., 2015).

Several members of the *Firmicutes* have been implicated as key cellulose degraders. Consistent with this pattern, enrichment of BD on lignocellulosic substrates led to a four to eightfold increase in the relative abundance of members from this phylum (**Figure 2A**). Most significantly, microbes belonging to the genus *Clostridium* and *Ruminococcus* were particularly enriched (**Figures 3B,C,E**), which is consistent with the importance of corresponding species to polysaccharide degradation (Tracy et al., 2012). Moreover, an uncultured lineage in order *OPB54* made up to 15% of the BD enrichments amended with cellulose (**Figure 3B**). Notably, *OPB54* was previously identified in low abundance in stillage biogas reactors that operated in high temperatures (Roske et al., 2014).

### Impact of Lignocellulosic Substrates on Microbial Communities Originating from Moose Rumen

Similar to the BD enrichments, phyla that were common to all enrichments derived from MR samples included *Firmicutes*, *Bacteroidetes*, *Chlorobi*, *Elusimicrobia*, and *Spirochaetes* (**Figure 2A**). In the case of samples from the MR enrichment cultures, all samples had a high relative abundance of *Firmicutes*, that was comparable between the inoculum (42%; **Figure 3A**) and enrichments amended with C (45%) and PH (58%; **Figures 3B,E**), but lower in enrichments on CL (13%) and higher in the enrichments on CT (93%) (**Figures 3C,D**). Most dramatically, the fraction of *Clostridium* species increased from 3% in the inoculum to 33% and 45% in C and PH enrichments, respectively (**Figures 3A,B,E**). By comparison, enrichments amended with CT were distinguished by over 45% of bacteria belonging to the *Lachnospiraceae* family (**Figure 3D**). The representation of this family decreased from 12% of the inoculum (**Figure 3A**) to less than 5% of other enrichments (**Figures 3B,C,E**). *Lachnospiraceae* members were previously identified in MR and foreguts of dromedary camels (Samsudin et al., 2011; Meehan and Beiko, 2014), but were not reported in reindeer gut (Pope et al., 2012).CT cultivations were further distinguished by an increase in the fraction of uncultured bacteria within the *Clostridia* class (**Figure 3D**), particularly from order *OPB54* as was observed for BD cultures enriched on C (increase from 0.1% in the inoculum to 6.3% in the enrichment) as well as unassigned genera within the *Lachnospiraceae* family (from 3 to 48% in the enrichment; **Figure 3A**).

In addition to the *Firmicutes*, members of the phylum *Chlorobi* increased from non-detectable levels in the inoculum to 17% and 27% of C and CL cultures (**Figures 3B,C**), respectively. As observed for corresponding enrichments of BD, this increase was mainly attributed to enrichment of bacteria belonging to class *SJA-28*. Members of the phylum *Elusimicrobia* (formerly *Termite Group 1*; *TG1*) were also enriched through growth on CL, from less than 1% in the inoculum to over 17% in the enrichment culture (**Figure 3C**). Notably, growth on other lignocellulosic amendments did not increase levels of *Elusimicrobia* members.

Whereas the relative abundances of *Firmicutes*, *Chlorobi*, and *Elusimicrobia* increased upon various lignocellulosic enrichments, the total fraction and species diversity of *Bacteroidetes* decreased from 23% in the inoculum to lower levels in the enrichment cultures (**Figure 2A**). Specifically, *BS11* (11%) and *Prevotella* (7%) in the MR sample became non-detectable after the enrichment process (**Figure 3A**), whereas an unassigned lineage under *Bacteroidales* was maintained after enrichment with C (4%), and increased upon CL (17%) and PH (22%; **Figures 3B,C,E**). In contrast, none of the *Bacteroidetes* species were detected in enrichment cultures amended with CT.

### Comparative Analysis of All Microbial Enrichments

As explained above, an underlying hypothesis of the enrichment study was that the relative abundance of microbes most relevant to lignocellulose conversion would increase by culturing the selected inocula on lignocellulosic substrates. In this way, we could also uncover microbial members that might encode specialized functions, including transformation potential inhibitory substances, such as tannic acid or lignosulphonate.

**FIGURE 4:**
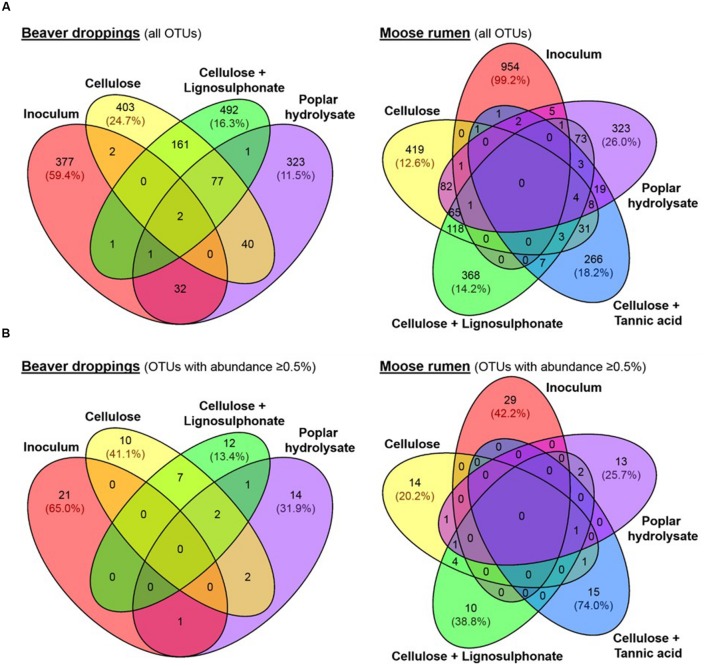
**Distribution of **(A)** all OTUs and **(B)** OTUs with relative abundances ≥ 0.5% in beaver dropping and moose rumen, and their corresponding enrichment cultures.** Abundances of represented OTUs are shown in brackets.

Consistent with our hypothesis, abundances of known lignocellulose degraders increased following amendment with selected lignocellulosic carbon sources and included microbial lineages previously identified in the termite hindgut, such as *Firmicutes*, *Proteobacteria*, *Bacteroidetes*, *Spirochaetes*, and *Elusimicrobia* (Huang et al., 2013), or the bovine rumen microbiome, such as *Chlorobi*, *Chloroflexi*, and *Fusobacteria* (Brulc et al., 2009). Moreover, UPGMA clustering of OTU sequences revealed convergence of microbial communities enriched with the same carbon source (**Figure 2A**); convergence of community composition was also revealed through UniFrac analysis (**Figure 2B**). Overall, microbial community compositions could be grouped into three main sub-clusters (**Figure 2A**), namely, (i) original inoculum, (ii) enrichment on C or CL, and (iii) enrichment on PH; the MR enrichment on CT formed a fourth, unique branch. Furthermore, the majority of OTUs present in BD and MR inocula were not detected in the corresponding enrichments (**Figure 4A**), this trend was even more obvious when a threshold of 0.5% abundance is applied (**Figure 4B**). Indeed, the most abundant OTUs in enrichments represented organisms that comprised less than 0.5% of all OTUs in the original inoculum (**Supplementary Data S2**). This demonstrates strong selection of microbial members for a given lignocellulose amendment, which was underscored by the few overlapping OTUs between enrichments on different lignocellulose amendments (**Figure 4B**). One notable exception was otu6272 (assigned to class *Gammaproteobacteria*), which represented nearly 8% of all OTUs in the BD inoculum and 14% of OTUs after enrichment on PH.

Core species were not identified among enrichments originating from the same inoculum (**Figures 4A,B**). Moreover, despite no shared OTUs between BD and MR inocula at a 0.5% abundance cut-off (**Figure 3A**), a high number of overlapping members constituting the shared dominant taxonomic lineages was observed between enrichments fed with the same lignocellulose carbon source (**Figures 3B,C,E**). For example, otu2346 assigned to the *Clostridium* genus was not detected in BD or MR inocula, but comprised a significant fraction of C enrichments (15–17%), CL enrichments (9%), and PH enrichments (7–49%; **Figures 3B,C,E**). Similarly, an OTU assigned to class *SJA-28* (otu225) was not observed in either inoculum, but represented 5–15% of both C enrichments and 20–28% of both CL enrichments (**Figures 3B,C**). Finally, otu4036 belonging to the order *Bacteroidales* was uniquely detected in enrichments established on PH (**Figure 3E**), where it comprised 5–10% of the bacterial community even though it was not detected in either inoculum or any other enrichment condition.

Despite the convergence of microbial communities enriched on the same lignocellulosic carbon source, unique lineages were also observed that reflect the impact of starting inocula. After enrichment of BD on Cl and PH, a *Ruminococcus* OTU (otu2378) represented 6% and 20% of corresponding microbial communities (**Figures 3C,E**). In contrast, otu2378 was not found in any MR enrichments. Similarly, the *Gammaproteobacteria* OTU (otu6272) and *W22* OTU (otu3890) identified in BD (**Figure 3A**) and corresponding enrichments on PH (**Figure 3E**) was not detected in MR samples or any of the derived enrichments.

**FIGURE 5:**
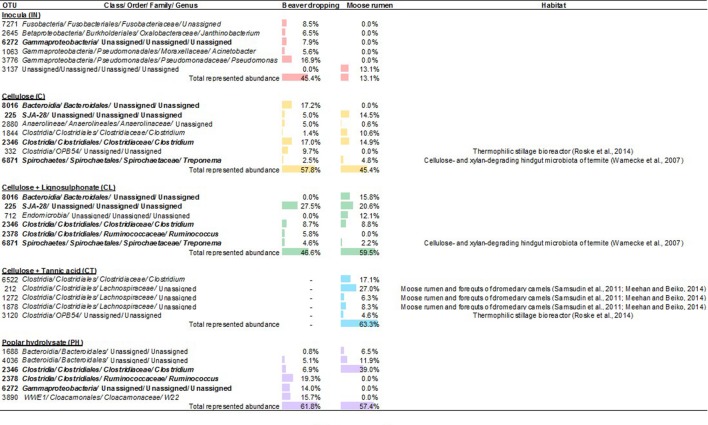
**Abundant OTUs from enrichment microcosms fed with various lignocellulosic carbon sources.** Abundances (≥ 4% in at least one sample) are indicated by the relative length of data bars, which are color coded to represent the inocula (red), and enrichment microcosms fed with cellulose (yellow), cellulose + lignosulphonate (green), cellulose + tannic acid (blue), and poplar hydrolysate (purple).

Correlations between microbial membership and lignocellulosic substrate also emerged by identifying key differences between communities resulting from the different amendments. For example, the abundance of class *SJA-28* in enrichments amended with CL was double that of enrichments amended with C. In contrast, the abundance of orders *OPB54* and *Clostridiales* consistently decreased upon amendment with CL compared to addition of C alone. **Figure 5** summarizes the specific OTUs that were enriched upon lignocellulosic amendment, as well as reported habitats of understudied lineages. In addition to these genera, class *Endomicrobia*, order *Bacteroidales*, family *Lachnospiraceae*, and genus *W22* represent additional sources of understudied microorganisms that could comprise unique enzymes and biochemical pathways relevant to lignocellulose conversion.

## Conclusion

Overall, enrichment of BD and MR on multiple lignocellulosic substrates led to the proliferation of recognized cellulolytic bacteria as well as unique lineages that were in low or undetectable abundances in corresponding inocula. These unassigned lineages were grouped in classes *SJA-28*, *Endomicrobia*, orders *Bacteroidales*, *OPB54* and family *Lachnospiraceae*, and comprised up to half of corresponding communities, warranting future investigation on their potential in lignocellulose degradation. The substrate-based convergence of microbial community compositions originating from BD and MR suggests that resulting communities have specialized to the amended carbon sources, and that corresponding microorganisms may encode distinct CAZymes that are particularly effective toward the given lignocellulosic carbon source. At the same time, microorganisms that were unique to specific enrichment conditions, such as *SJA-28* in enrichments amended with CL and bacteria from order *OPB54* or *Lachnospiraceae* family in enrichments amended with CT, may comprise specialized catabolic activities relevant to pretreatment and detoxification of wood hydrolysates (Lopez et al., 2004); metagenomic analyses are now underway to investigate these predictions.

## Author Contributions

MW performed the sequence analyses and data interpretation, and compiled the manuscript. WW maintained the enrichment cultures, prepared DNA samples for sequencing, and contributed to data interpretation. ML collected the environmental samples and established the enrichment cultures. MC contributed to data interpretation. EM and EAE conceived and coordinated the study. All authors contributed to the revision of manuscript and approved the final version.

## Conflict of Interest Statement

The authors declare that the research was conducted in the absence of any commercial or financial relationships that could be construed as a potential conflict of interest.

## Footnotes

1 http://www.454.com

2 http://www.r-project.org

## Abbreviations

BD: beaver droppings
C: cellulose
CAZyme: carbohydrate-active enzyme
CL: cellulose + lignosulphonate
COD: chemical oxygen demand
CT: cellulose + tannic acid
MR: moose rumen
OTU: operational taxonomic unit
PH: poplar hydrolysate
PCoA: principal coordinate analysis
UPGMA: unweighted pair group method with arithmetic mean
