# Supplementary material for: Substrate-Driven Convergence of the Microbial Community in Lignocellulose-Amended Enrichments of Gut Microflora from the Canadian Beaver (Castor canadensis) and North American Moose (Alces americanus)
Source: Front Microbiol. 2016 Jun 21;7:961. doi: 10.3389/fmicb.2016.00961 (PMC4914502; doi:10.3389/fmicb.2016.00961)
Supplement: Supplementary file 3 [file Presentation_1.PDF]

Supplementary information

Substrate-driven convergence of the microbial community in lignocellulose-amended enrichments of gut microflora from the Canadian beaver (*Castor canadensis*) and North American moose (*Alces americanus*)

Mabel T. Wong, Weijun Wang, Michael Lacourt, Marie Couturier, Elizabeth A. Edwards, Emma R. Master*

*Correspondence: Emma R. Master (emma.master@utoronto.ca)

1. Supplemental methods

Calculation of stoichiometric maximum biogas volume per mg COD carbohydrates:

According to Buswell's equation (Symons and Buswell, 1933),

$C_nH_aO_bN_d + (n-a/4-b/c+3d/4)\ H_2O \rightarrow (n/2+a/8-b/4-3d/8)\ CH_4 + (n/2-a/8+b/4+3d/8)\ CO_2 + dNH_3$

Using glucose as an example for carbohydrate, we have $C_6H_{12}O_6 \rightarrow 3\ CH_4 + 3\ CO_2$. Assuming ideal conditions and no dissolution of CO_2 in the medium, 1 mole of glucose yields 6 mole of gases. Using the ideal gas law (PV = nRT or V = nRT÷P), 1 mole of glucose yields 6 mol × 298 K × 0.082057 L atm K^{-1} mol^{-1} ÷ 1 atm = 146.7 L biogas, or 1 mg of glucose yields 0.8142 ml biogas. Consider the oxidation of glucose: $C_6H_{12}O_6 + 6\ O_2 \rightarrow 6\ CO_2 + 6\ H_2O$, the chemical oxygen demand (COD) of glucose = 6 × 32 g/mol O_2 ÷ 180.1559 g/mol glucose = 1.066 g O_2/g glucose. Therefore, the stoichiometric maximum biogas yield is 0.8142 ml biogas/mg glucose ÷ 1.066 g O_2/g glucose = 0.764 ml biogas/mg COD.

Consider sodium lignosulphonate ($C_{20}H_{24}Na_2O_{10}S_2$) (National Center for Biotechnology Information, 2016b), 1 mole of this substrate yields 489.1 L biogas containing 10.5 mole of CH_4

and 9.5 mole of CO_2. Based on the measured COD of sodium lignosulphonate (Table S1), the stoichiometric maximum biogas yield is 0.58 ml biogas/mg COD. Similarly, 1 mole of tannic acid ($C_{76}H_{52}O_{46}$) (National Center for Biotechnology Information, 2016a) generates 1858.4 L biogas containing 33 moles of CH_4 and 43 mole of CO_2 based on stoichiometry, and the calculated maximum biogas yield is 0.90 ml biogas/mg COD using the measured COD of tannic acid (Table S2). In proportion of the substrates added at each enrichment phase, the stoichiometric maximum biogas yields (ranged from 0.69 to 0.82 ml biogas/mg COD substrate) were derived (Table S2). Actual yield may differ owing to factors such as limited biodegradability of compounds and solubility of CO_2 in liquid.

2. Supplementary Data

Supplementary Data 1 Summarized relative abundances of microbial lineages in beaver dropping, moose rumen and their enrichment microcosms.

Supplementary Data 2 Taxonomic assignment of the OTUs defined at 97% nucleotide identity.

3. Supplementary Figure and Tables

3.1 Supplementary Figure

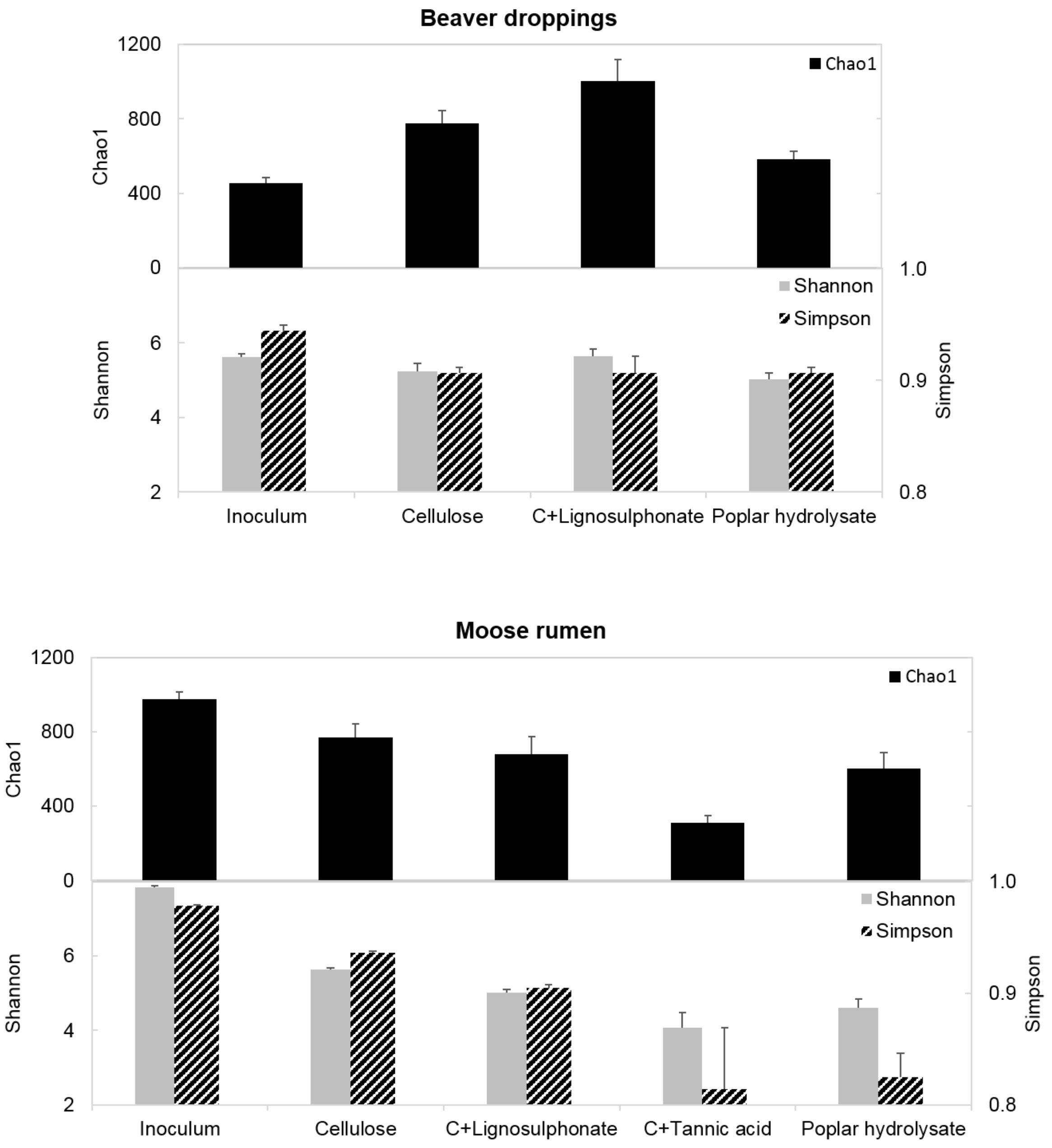

Figure S1 Diversity indices of inocula and corresponding enrichments amended with various lignocellulosic substrates. Error bars indicate standard deviation; n=3.

3.2 Supplementary Tables

Table S1 Measured chemical oxygen demand (COD) of enrichment substrates (± standard deviation).

Enrichment substrate	COD content (g COD/g substrate)
Cellulose	1.22 ± 0.12
Sodium lignosulphonate	1.54 ± 0.11
Tannic acid	1.22 ± 0.00
Poplar hydrolysate	0.93 ± 0.30

Table S2 Lignocellulosic amendments for enrichment microcosms and stoichiometric maximum biogas yields.

Enrichment	Cellulose (C)		Lignosulphonate (L), Tannic acid (T), or Poplar hydrolysate (PH)		Total COD (mgCOD)	Estimated stoichiometric maximum biogas yield (ml biogas/mg COD substrate)
	mg per bottle	mgCOD per bottle	mg per bottle	mgCOD per bottle		
**Phase 1**						
C	28.4	33.7	-	-	33.7	0.76
CL	22.8	27	5.3	8.1	35.1	0.72
CT	22.8	27	17.7	22	49	0.82
PH	-	-	28.4	26.6	26.6	-
**Phase 2-3**						
C	142.2	168.5	-	-	168.5	0.76
CL	113.8	134.9	26.3	40.5	175.4	0.72
CT	136.5	161.8	17.7	22	183.8	0.78
PH	-	-	142.2	132.8	132.8	-
**Phase 4**						
C	113.8	134.9	-	-	134.9	0.76
CL	113.8	134.9	26.3	40.5	175.4	0.72
CT	113.8	134.9	88.5	109.9	244.8	0.82
PH	-	-	136.5	127.5	127.5	-
**Phase 5-10**						
C	125	152.5	-	-	134.9	0.76
CL	125	152.5	90	109.8	262.3	0.69
CT	125	152.5	26.3	40.5	193	0.79
PH	-	-	136	126.5	126.5	-

Table S3 Dates and duration of microcosm enrichment phases 1 to 4, 7, and 9.

	Inocula	Beaver dropping				Moose rumen			
	**Substrates**	**C**	**CL**	**CT**	**PH**	**C**	**CL**	**CT**	**PH**
**Phase 1**	Start date[1]	10-02-12	10-02-12	10-02-12	10-02-12	09-10-13	09-10-13	09-10-13	09-10-13
	Duration (d)	124	124	124	124	201	201	201	201
**Phase 2**	Start date	10-07-23	10-07-23	10-07-23	10-07-23	10-08-12	10-08-12	10-08-12	10-08-12
	Duration (d)	55	55	55	55	62	62	62	62
**Phase 3**	Start date	10-09-30	10-09-30	10-09-30	10-09-30	10-11-17	10-11-17	10-11-17	10-11-17
	Duration (d)	90	90	90	90	58	58	58	58
**Phase 4**	Start date	11-01-06	11-01-06	11-01-06	11-01-06	11-01-15	11-01-15	11-01-15	11-01-15
	Duration (d)	30	30	30	30	30	30	30	30
**Phase 7**	Start date	11-12-05	11-12-05	11-12-05	11-12-05	11-12-05	11-12-05	11-12-05	11-12-05
	Duration (d)	259	176	259	259	259	176	259	259
**Phase 9**	Start date	12-11-06	12-11-12	12-11-01	12-10-20	12-11-06	12-11-08	12-11-01	12-10-20
	Duration (d)	78	71	82	94	78	75	82	94

[1] Dates are displayed as YY-MM-DD.

Table S4 Multiplex barcodes, DNA concentration, and numbers of reads for amplicon samples prepared from beaver dropping, moose rumen and their enrichment microcosms.

Enrichment condition	Multiplex barcode	Amplicon DNA concentration (µg/ml)	Number of reads
**Beaver dropping**			
Inoculum	TAGTGTAGAT	31.7	4504
	TCGCACTAGT	38.2	5116
	TCTATACTAT	43.4	5213
Cellulose	ACATACGCGT	138	7471
	ACGCGAGTAT	116.3	7828
	ACTACTATGT	168.9	8312
Cellulose+Lignosulphonate	TACGAGTATG	158.9	9475
	TAGAGACGAG	133.1	8191
	TCGTCGCTCG	90.3	5921
Cellulose+Tannic acid	ATAGAGTACT	29.1	-
	CACGCTACGT	30.5	-
Poplar hydrolysate	AGTACGCTAT	99.1	8699
	ATAGAGTACT	116.4	9232
	CACGCTACGT	51.2	4441
**Moose rumen**			
Inoculum	CGACGTGACT	34.2	3198
	TACACGTGAT	25.8	3444
	TACGCTGTCT	20.1	5339
Cellulose	TGTACTACTC	154.9	8051
	ACGACTACAG	133.8	6972
	CGTAGACTAG	163.6	8523
Cellulose+Lignosulphonate	TGATACGTCT	154.3	8694
	CGAGAGATAC	116.9	6378
	TCTACGTAGC	129.4	7100
Cellulose+Tannic acid	AGCGTCGTCT	23.6	9144
	AGTACGCTAT	65.2	9128
Poplar hydrolysate	CAGTAGACGT	85.2	8522
	TACACGTGAT	68.2	5739
	TACGCTGTCT	100.5	8673

Table S5 Statistics of 16S rRNA pyrotag sequencing runs.

Sample	Total number of reads	Number of OTUs	Number of OTUs with abundance ≥0.5% (sum of represented abundances)
**Beaver droppings**			
Inoculum	14564	415	22 (72.9%)
Cellulose	23268	685	21 (77.6%)
Cellulose+Lignosulphonate	23024	840	23 (70.2%)
Cellulose+Tannic acid[1]	12	-	-
Poplar hydrolysate	22077	581	21 (77.9%)
**Moose rumen**			
Inoculum	11291	967	29 (42.2%)
Cellulose	23091	734	22 (73.3%)
Cellulose+Lignosulphonate	21869	644	18 (76.4%)
Cellulose+Tannic acid	18003	346	17 (80.5%)
Poplar hydrolysate	22614	588	18 (76.0%)

[1] Dataset for beaver droppings enriched on cellulose plus tannic acid was removed from downstream analysis due to low number of reads (less than 15).

Reference

National Center for Biotechnology Information (2016a). *PubChem Compound Database; CID=16129778* [Online]. Available: https://pubchem.ncbi.nlm.nih.gov/compound/tannic_acid [Accessed 24 April 2016].

National Center for Biotechnology Information (2016b). *PubChem Compound Database; CID=25113562* [Online]. Available: https://pubchem.ncbi.nlm.nih.gov/compound/25113562 [Accessed 24 April 2016].

Symons, G.E., and Buswell, A.M. (1933). The methane fermentation of carbohydrates. *J Am Chem Soc* 55**:**2028-2036. doi: Doi 10.1021/Ja01332a039.
